# Pharmacodynamics, Population Dynamics, and the Evolution of Persistence in *Staphylococcus aureus*

**DOI:** 10.1371/journal.pgen.1003123

**Published:** 2013-01-03

**Authors:** Paul J. T. Johnson, Bruce R. Levin

**Affiliations:** Department of Biology, Emory University, Atlanta, Georgia, United States of America; Fred Hutchinson Cancer Research Center, United States of America

## Abstract

When growing populations of bacteria are confronted with bactericidal antibiotics, the vast majority of cells are killed, but subpopulations of genetically susceptible but phenotypically resistant bacteria survive. In accord with the prevailing view, these “persisters” are non- or slowly dividing cells randomly generated from the dominant population. Antibiotics enrich populations for pre-existing persisters but play no role in their generation. The results of recent studies with *Escherichia coli* suggest that at least one antibiotic, ciprofloxacin, can contribute to the generation of persisters. To more generally elucidate the role of antibiotics in the generation of and selection for persisters and the nature of persistence in general, we use mathematical models and experiments with *Staphylococcus aureus* (Newman) and the antibiotics ciprofloxacin, gentamicin, vancomycin, and oxacillin. Our results indicate that the level of persistence varies among these drugs and their concentrations, and there is considerable variation in this level among independent cultures and mixtures of independent cultures. A model that assumes that the rate of production of persisters is low and persisters grow slowly in the presence of antibiotics can account for these observations. As predicted by this model, pre-treatment with sub-MIC concentrations of antibiotics substantially increases the level of persistence to drugs other than those with which the population is pre-treated. Collectively, the results of this jointly theoretical and experimental study along with other observations support the hypothesis that persistence is the product of many different kinds of errors in cell replication that result in transient periods of non-replication and/or slowed metabolism by individual cells in growing populations. This Persistence as Stuff Happens (PaSH) hypothesis can account for the ubiquity of this phenomenon. Like mutation, persistence is inevitable rather than an evolved character. What evolved and have been identified are genes and processes that affect the frequency of persisters.

## Author Summary

Because of its importance to therapy, a great deal of effort has been devoted to understanding the mechanisms responsible for and the genetic basis of persistence in inherently susceptible but phenotypically antibiotic-resistant subpopulations of bacteria. Much of this research is based on the premise that persisters are produced at random from the susceptible population and the antibiotics used to detect them play no role in their generation. The results of this jointly theoretical and experimental study are inconsistent with this hypothesis. These results, along with observations reported by other investigators, including the failure to find bacteria that do not produce persisters but an abundance of genes modifying their frequency, support the hypothesis that there are many mechanisms responsible for persistence. Based on these collective theoretical and experimental results, along with evolutionary considerations, we postulate that persistence is analogous to mutation. It is an inevitable product of errors and glitches in cell replication and metabolism rather than an evolved character.

## Introduction

While it is convenient to consider genetically identical populations of bacteria as collections of physiologically homogeneous cells, they are commonly composed of phenotypically different subpopulations. Usually by some form of stochastic switch, during the course of growth or at stationary phase, bacteria of one phenotype produce cells of different phenotypes [1]–[6]. From the practical perspective of antibiotic treatment, the most important of these phenotypically distinct subpopulations are the persistent cells. As first reported by John Bigger [7], when growing populations of bacteria are confronted with bactericidal antibiotics, the majority of cells are killed but a minority survive. Upon re-culture, these surviving bacteria are as sensitive to antibiotics as the cells from whence they were derived. And, when exposed to bactericidal antibiotics, they too produced minority populations of survivors. Bigger called these phenotypically resistant but genetically susceptible subpopulations of bacteria “persisters”.

In his original 1944 studies, Bigger used *Staphylococcus pyogenes* (now *Staphylococcus aureus*) and the beta-lactam antibiotic penicillin. Since then, persistence has been demonstrated for a number of different species of bacteria with many classes of antibiotics [8]–[15]. Indeed, persistence has been suggested to be a universal character, not only among the bacteria [16] but also fungi [17], [18], the cells responsible for neoplasms [19] and doubtless other somatic cells. Although it is not absolutely clear how important persister subpopulations are to the course of antibiotic treatment in human patients, at least in theory persisters can prolong the course therapy, prevent clearance and promote the generation and ascent of bacteria with heritable resistance [20], [21]. There is in fact recent clinical evidence for persistence retarding the rate of, and possibly preventing the clearance of bacterial infections in antibiotic treated humans [22], [23].

What are persisters? In accord with the prevailing view, bacterial persisters are subpopulations of cells that are refractory to antibiotics because they are either not dividing or they are dividing at very low rates [16], [24], [25], [26]. In this perspective, one class of persisters are generated at random by a stochastic switch from non-persisters during exponential growth, remain in that state for some time and then revert to the non-persister state [24], [25]. It has been proposed that persisters are also generated at stationary phase as, upon transfer to fresh medium, they take longer to come out of lag (“wake up”) than non-persisters [27]. Further work has shown that during the first 1.5 hours following inoculation into fresh media, the class of non-growing persister cells generated during stationary phase are not yet completely dormant and not yet refractory to antibiotic treatment but rather complete this transition during this time period [28]. Thus, by killing the majority population of replicating cells, antibiotics increase the relative frequency of these phenotypically resistant but genetically susceptible bacteria, i.e. antibiotics reveal the existence of persister subpopulations, but have no role in their generation. A recent report by Dörr and colleagues questions the generality of this perspective on the role of antibiotics in the generation of persisters [29]. In their study with *E coli*, pre-exposure to the fluoroquinolone ciprofloxacin increased fraction of cells surviving exposure to bactericidal concentrations of this drug (also see [30]).

In this investigation, we use mathematical models, computer simulations and experiments with *Staphylococcus aureus* and antibiotics of four classes to determine the effects of different antibiotics and their concentrations on the level of persistence and more broadly elucidate the contribution of these drugs to the formation of persisters. We interpret the results of these experiments to be inconsistent with the hypothesis that persisters are a unique, or even very few types of subpopulations that are generated at random, and that bactericidal antibiotics only reveal their existence by killing growing cells. Our results provide evidence that the singular observation that pre-exposure of *E. coli* to ciprofloxacin increases the frequency of persisters in generally applicable. We show that it not only obtains with a very different species of bacteria, but also with antibiotics of different classes. Based on these theoretical and experimental results, the results of other studies of persistence, and evolutionary considerations we postulate that persisters are: (i) the product of many different of errors and/or processes that result in transient periods of inhibition of the cell replication cycle of individual metabolizing bacteria and/or affect the amount of time required for the transition from stationary phase to replication (the lag), and (ii) the rates at which these processes occur are influenced by environmental stresses including the presence of antibiotics. We propose that persistence is analogous to mutation, an inevitable rather than an evolved character.

## Results

### Theoretical considerations

We open this report with a quantitative straw person. We use a simple mathematical model of to illustrate what would be anticipated in our experiments if persisters are generated at random at a rate that is independent of the nature and quantity of antibiotics that select for these phenotypically resistant cells, the null hypothesis. In this model, there are three populations of bacteria, one that is replicating at a high rate and two that are not replicating, persisters, with densities and designations, *N*, *P_E_* and *P_S_*, respectively. As shown by Balaban and colleagues [24], we assume that these persisters are of two types: 1- those that are produced at stationary phase, *P_S_*, and those that are produced when the cells are growing exponentially, *P_E_*. These persister cells are generated from the *N* population at maximum rates, *f_s_* and *f_e_*, per cell per hour and return to the *N* state at maximum rates *g*_s_ and *g_e_*, per cell per hour. To distinguish between growing and stationary phase, we assume a logistic function (*1-N_T_/K*) where *N_T_* is the total density of bacteria and *K* the saturation (stationary phase) density, where *N_T_≤K*. The rate of growth of the bacteria, the rate of production of persisters from growing cells *N→P_E_*, and the rate of return from the persister to the growing state, *P_S_→N* and *P_E_→N* is proportional to (*1-N_T_/K*). The idea being that these rates are greater when the population is further from stationary phase and these processes cease when the population is at stationary phase, *N_T_ = K*. The rate of production of stationary phase persisters, *N→P_S_*, on the other hand is proportional to *N_T_/K* and thereby increases as the population approaches stationary phase and continues during stationary phase.

For the pharmacodynamics of the antibiotic, and the *N* population we use a Hill function [31],
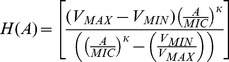
where *A* is the concentration of the antibiotic, *V_MAX_* the maximum growth rate of the bacteria, *V_MIN_* the minimum growth rate, (*V_MIN_*≤0), the minimum inhibitory concentration (*MIC*) of the antibiotic and *k* the hill coefficient, a shape parameter. With these definitions and assumptions, the rates of change in the densities of these three states of bacteria are given by
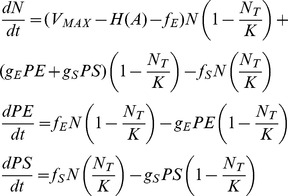
where *N_T_ = N+PE+PS*

To illustrate the properties of this model, we use a finite step size Euler method to numerically solve these differential equations. For these computer simulations, the generation and loss of persisters are stochastic processes, which we simulate by a Monte Carlo process. For example, when the rectangularly distributed random number, *r (0≤r≤1)* is less than the product of the f_e_(1-N_T_/K) *N*Δt, a single individual of the *P_E_* persister population is generated and a single individual of the *N* population is lost. The step sizes of these simulations, *ΔT*, are chosen so that during any finite step, the probability of generating a persister is less than 1. These computer simulations were programmed in Berkeley Madonna. Copies of this program and instructions for its used are available at www.eclf.net.

#### Predictions

**Figure 1:**
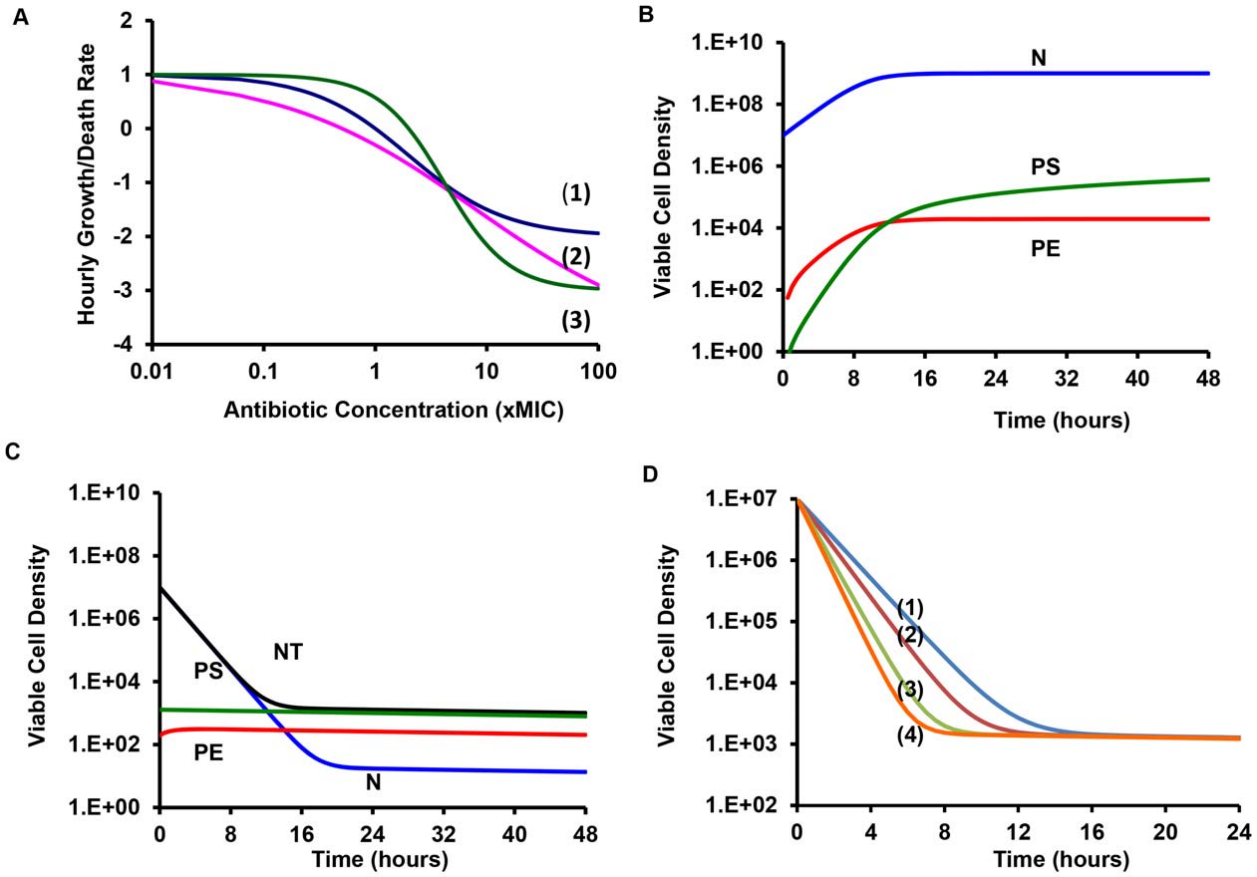
Pharmacodynamics and anticipated population dynamics of treatment. (A) Antibiotic-dependent growth/death rates. Hill function *V_MAX_* = 1 for all, the values of *V_MIN_, MIC, κ* are respectively (1) −1: 1: 1, (2) −3, 1, 1, (3) −5.0, 0.5, 0.5. (B) Changes in viable cell density in the absence of antibiotics, *K* = 2×10^9^, *f_E_* = 10^−5^, *g_S_* = 10^−3^, *g_E_* = 10^−3^ per persister cell per hour. (C) Changes in viable cell density with 10 µg/ml of an antibiotic and the Hill function parameters in (A) line (1). The rest of the parameters in this and the following figure were the same as those in 1B. In this, and the following figure, the initial density of cells was 2×10^7^ and the initial densities of *P_S_* and *P_E_* were 1/100 of that generated during the 24 hours before the start of the run. (D) Changes in total viable cell density, *N_T_* following exposure to antibiotics with different Hill function parameters and/or drug concentration. Lines (1) and (2) – Hill function in (A) line (1) with respectively 10 and 100 µg/ml of the antibiotic, Line (3) Hill function parameters in (A) line (2) and 100 µg/ml of the antibiotic, Line (4) Hill function parameters in (A) line (3) and 100 µg/ml of the antibiotic.

In Figure 1A, we present the Hill function antibiotic concentration – dependent growth/death rates for bacteria confronting bactericidal drugs with different combinations of *MICs*, *V_MIN_s* and *κs*. In Figure 1B, we follow the dynamics of change in density of normal, *N*, bacteria and bacteria of the stationary and exponential phase persister states, *P_E_* and *P_S_*, respectively. Initially, the population in this simulation consisted solely of 10^3^ *N* cells per ml. Within short order, the *N* population reaches the saturation level *K*. During the course of its ascent, persisters of the *P_S_* and *P_E_* populations also accumulate. Although the density of the persisters generated during exponential growth, *P_E_*, stopped increasing when the *N* population at large *N_T_*, enters stationary phase, *N_T_ = K*, the stationary phase persister population *P_S_*, continued to ascend.

The antibiotic kills the sensitive *N* cells but the initial presence and continuous production of the *P_S_* and *P_E_* persister cells prevents the total population from being cleared (Figure 1C). Because of reversion from the persisters to the *N* state, the sensitive population remains present. With these parameters the total population of bacteria slowly wanes as time proceeds. In Figure 1D we consider the effect of antibiotics with different properties (Hill function parameters) and concentrations on the changes in total density of viable cells, *N_T_*. There are substantial differences among drugs and concentrations in the rates at which the bacteria are killed. On the other hand, there are only modest differences in the viable cell density (primarily the density of persisters) after the population levels off. At 24 hours, the average viable cell density for 10 independent simulation runs with the most rapidly killing treatment, line (4) in Figure 1D, was 4.2×10^2^ whilst that for treatment with the lowest rate of killing, line (1) in Figure 1D, was 5.9×10^2^. The ratio of the standard deviation to the mean density of viable cells at 24 hours among the 10 runs was relatively low, on the order of 0.02.

### Experiments

#### MIC estimates

In Table 1, we list the estimated MICs for the *S. aureus*- antibiotic combination used in the following experiments. Considering the factor of two limitation of the CLSI serial dilution protocol, the MIC estimated by both of these methods are coincident.

**Table 1:** Estimated MICs for *S. aureus* (Newman) by serial dilution and calculated from the Hill functions.

Antibiotic	CLSI MIC	Hill function MIC
Gentamicin	1.5 µg/ml	0.72 µg/ml
Vancomycin	2.0 µg/ml	1.05 µg/ml
Ciprofloxacin	0.5 µg/ml	0.38 µg/ml
Oxacillin	0.2 µg/ml	0.22 µg/ml

#### Short-term time kill—persistence level experiments

**Figure 2:**
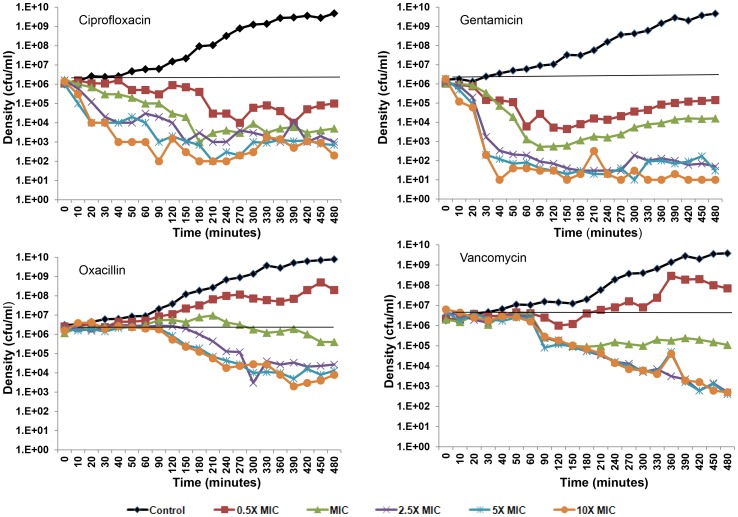
Short-term time kill experiments. Changes in viable cell density for *S. aureus* Newman cultures treated with varying concentrations (0.5× MIC, MIC, 2.5× MIC, 5× MIC, and 10× MIC) of ciprofloxacin, gentamicin, oxacillin, and vancomycin.

In Figure 2, we present the results of the *S. aureus* time kill experiments run over eight hours with frequent estimates of viable cell density. Antibiotic mediated killing starts almost immediately after ciprofloxacin and gentamicin are added to the cultures, but does not commence for about an hour after oxacillin and vancomycin are added (2 hours after the overnight cultures were diluted into fresh medium). Persistence can be seen in all cultures with above MIC concentrations of these drugs. Moreover, for ciprofloxacin and gentamicin some killing and persistence are observed for the cultures exposed to 0.5× MIC as well as supra-MIC concentrations during these eight hours. These data also suggest that the viable cell density of the persistent population may vary with the drugs and possibly their concentrations. The persistence level of cells exposed to gentamicin appears to be an order of magnitude, or more, lower than that of the other drugs.

To test the hypothesis that that the cells surviving antibiotic treatment, the persisters, are only transiently refractory to antibiotics (phenotypically resistant), we conducted a second round of short term time kill experiments initiated with overnight cultures derived colonies surviving the first encounter with antibiotics. Upon re-exposure to the same concentration of each antibiotic that they were exposed to during the first time kill experiment, the persistence levels and general killing dynamics were recapitulated for all supra-MIC concentrations of antibiotics (Text S1).

#### Longer-term time kill—persistence level experiments

**Figure 3:**
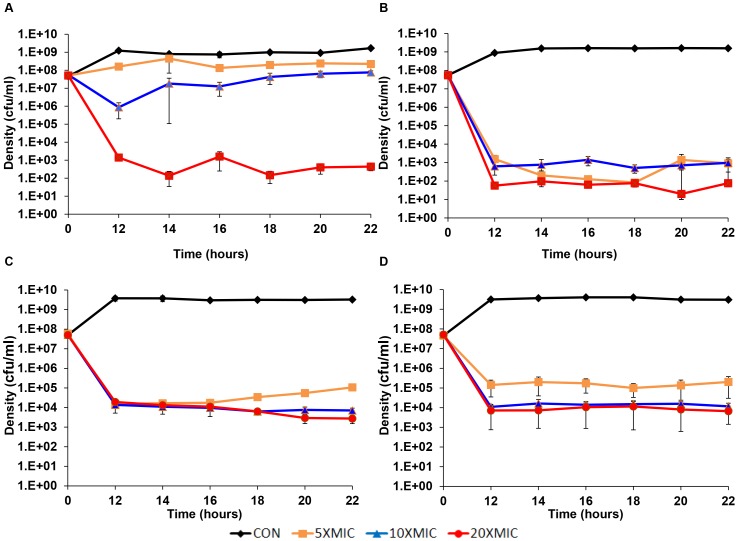
Longer-term time kill experiments. Changes in viable cell density, means and standard errors (bars), for three independent cultures of *S. aureus* each exposed to different concentrations (5× MIC, 10× MIC and 20× MIC) of four antibiotics: (A) Ciprofloxacin, (B) Gentamicin, (C) Oxacillin and (D) Vancomycin.

Experiments were performed to determine the relationship between the different antibiotics, their concentrations and the levels of persistence over a longer period of exposure. In the first of these longer-term persistence experiments, cultures were incubated for 12 hours with different concentrations of the four antibiotics used previously before sampling. Starting at the 12^th^ hour, viable cell densities were estimated every two hours for the following 10 hours (Figure 3). This experiment differed from the short-term time kill experiment (Figure 2) in two ways. First, the initial density of bacteria in these longer-term cultures was greater than that we used in the short-term experiments and we only considered above MIC concentrations of the drugs. Second, we also performed three independent replicas each initiated from separate overnight cultures. It should be noted, that while in these experiments antibiotics were not added until an hour after inoculation, effectively the same final level of persistence obtains when antibiotics are added immediately (data not shown).

The existence of a persistent subpopulation is apparent for bacteria exposed to all four drugs. For the higher concentrations of these drugs, the level of persistence seems relatively constant between 12 and 22 hours. With higher concentrations of these antibiotics, the level of persistence of the cultures treated with gentamicin and ciprofloxacin appear to be lower than that of vancomycin and oxacillin. For vancomycin, there seems to be a slight concentration effect. A concentration effect also seems to be the case for the level of persistence for oxacillin and ciprofloxacin, but see the following.

To test for inherited resistance, colonies were taken at 22 hours from these cultures, suspended in MHII broth and tested for resistance by disk diffusion assays. The only resistance observed in these assays was for the colonies recovered from the experiments with 5× and 10× MIC ciprofloxacin. Thus the seemingly higher level of persistence for these lower concentrations of this fluoroquinolone can be attributed to the evolution of bacteria that were inherently resistant to this drug. In the case of oxacillin, the higher level of persistence at lowest concentration (5× MIC) cannot be attributed to resistance, but rather appears to be due to a decline in the effective concentration of this drug. Evidence for this are the results of a bioassay, where we estimated the MIC of cell-free supernatants recovered from the 5× MIC oxacillin cultures. During the course of the 24 hours, the apparent MIC of this supernatant increased 4 fold. No significant increase in the apparent MIC was noted for the cultures with 10× and 20× MIC of oxacillin.

**Figure 4:**
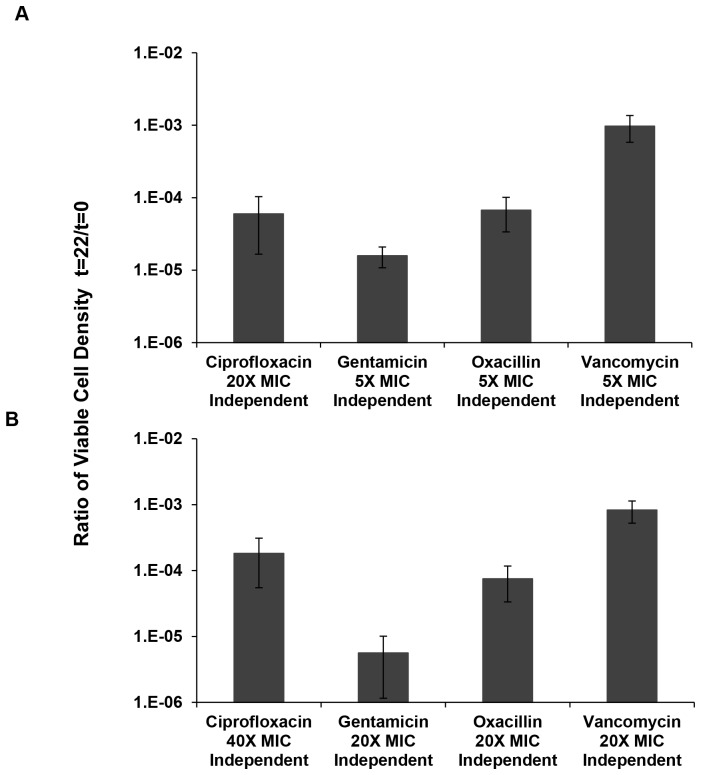
Relative survival after 22 hours of exposure to antibiotics. Ratio of viable cell densities (22 hours/0 hours) following treatment of 10 independent cultures of *S. aureus* Newman (for oxacillin and vancomycin only 9 samples are used for the calculation as we removed the extreme high numbers from each of them). (A) Mean and standard deviation of viable cell density in cultures containing “low” multiplicities of MIC for ciprofloxacin, gentamicin, oxacillin and vancomycin. (B) Mean and standard deviation of viable cell density in cultures containing “high” multiplicities of MIC for ciprofloxacin, gentamicin, oxacillin and vancomycin.

Antibiotic and concentration effects on the level of persistence: In an effort to better determine the effects of the nature and concentration of antibiotics on the level of persistence for two concentrations of each drug, we performed two-point time kill experiments with 10 independent replicas. For each experiment, we prepared 10 independent starting cultures, initiated with less than 5×10^6^ cells per ml, and at times 0 and 22 hours we estimated the densities of viable cells in the cultures from CFU data. In Figure 4, we present the results of these experiments.

Using a one-factor analysis of variance, we tested for differences in the fraction of cells surviving after 22 hours of exposure to 5× and 20× MIC of the different antibiotics relative to the initial inoculum, the persistence fraction. We did not do a 5× MIC experiment for ciprofloxacin because it was already clear that the fraction of surviving cells at this concentration was substantially greater than the other three drugs (Figure 3). Instead, we used 20× and 40× MIC in the two-point time kill experiments with this antibiotic. If we include the oxacillin survivors, the differences in viable cell densities for the 5× MIC comparison (gentamicin, oxacillin and vancomycin) were highly significant (F(2,26) = 25.6, p<0.005). If we remove oxacillin from this comparison, the between drug difference in density of persisters remains significant but less so, p<0.04 (t-test with unequal variances). For the 20× MIC comparison there were no significant differences in the level of persistence of cells exposed to the different antibiotics, F(3,34) = 1.4, p∼0.23).

For three out of the four drugs considered, gentamicin, oxacillin and vancomycin, there were significant differences between the 5× and 20× MIC survival, p<0.002 for a 2-tailed t-test with unequal variances. The level of persisters for the 20× and 40× MIC ciprofloxacin experiments were not significant, p∼0.95. But as also seen in Figure 2, at both 5× and 10× MIC of ciprofloxacin there were substantially greater numbers of surviving cells at 22 hours than at 20× MIC of ciprofloxacin.

#### The variance

**Figure 5:**
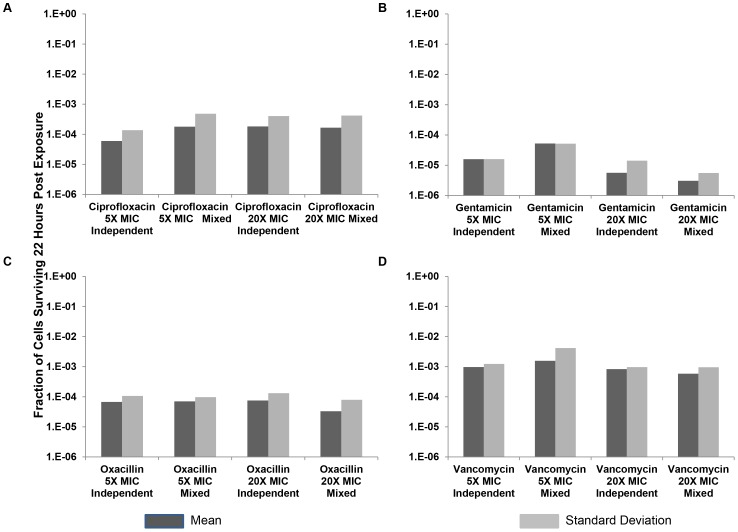
Relative survival after 22 hours of exposure to antibiotics. Means and standard deviations of the fraction of persisters following treatment for 10 independent cultures, and 10 cultures derived from mixtures of equal amounts of the 10 independent cultures, with either 5× MIC or 20× MIC of four antibiotics. (A) Ciprofloxacin, (B) Gentamicin, (C) Oxacillin, and (D) Vancomycin. In these experiments, the initial density of cells exposed to the antibiotics was on the order of 10^6^ to reduce the incidence of mutants for ciprofloxacin (none were observed).

Particularly striking in these data is the large variance in the number of persisters generated among cultures exposed to the same antibiotic. Included among these samples are cultures for which no cells were detected at minimum dilution (1/10) and cultures with more than 100 colonies. To illustrate the magnitude of this between-culture variation, we plot the standard deviation as well as the mean fraction of surviving cells for the different antibiotics in 10 independent cultures treated with different antibiotics and concentrations there-of (Figure 5).

To determine whether the variation in number of cells surviving exposure to the antibiotics is generated while the bacteria are growing or after they encountered the antibiotic, we performed fluctuation experiments similar to those Luria and Delbruck used to test whether mutations occur before exposure to the selective agent [36]. For this we compared the relative survival of antibiotic-exposed cells among independent cultures and cultures derived from equal volume mixtures of independent cultures. If the antibiotics only selected for pre-existing persisters, the variance in relative frequency of persisters would be substantially greater among independent cultures than the mixtures of independent cultures. The protocol for this experiment was the same as that for the experiments depicted in Figure 4. However, for the mixed cultures, equal volumes of cells from the overnight independent cultures were mixed together prior to dilution in fresh media. Following this mixing, 10 replica's were prepared in fresh media in parallel with the 10 independent cultures prepared in fresh media, allowed to grow for 1 hour, and then were incubated with either 5× or 20× MIC of their respective antibiotic for 22 hours.

As seen in Figure 5, the variation in the between culture number of persisters among the cultures that were inoculated with equal volume mixtures of individual cultures was similar to that among those inoculated with the independent cultures. This is what would be anticipated if antibiotics played a role in determining the number of persisters rather than just enriched these cultures for pre-existing persisters.

To determine the extent to which the above-described variance in the fraction of surviving cells can be attributed to variation in the ability to form colonies by the antibiotic exposed cells (plating efficiency), we plated 10 independent samples from single cultures of bacteria exposed to each of the four antibiotics. The ratios of the standard deviations to the means are respectively, 0.33, 0.10, 0.26 and 0.30 for ciprofloxacin, vancomycin, oxacillin and gentamicin. The extent of variation in the plating efficiency of antibiotic treated cultures is greater than that for the antibiotic-free control. This suggests that antibiotic exposure effects on plating efficiency accounts for some of the large variance among independent persister samples. However, the magnitude of this plating efficiency effect is far less than that which could account for the large variation among independent cultures seen in Figure 5.

#### A hypothesis

How do we account for the large variance in the fraction of persisters among independent and mixed cultures? As noted in the opening theoretical consideration, if antibiotics only enrich populations for existing persisters the between culture variance in the number of persisters is anticipated to be small (less than 5% of the mean) and we would not expect some cultures not to produce any persisters while others would produce many. One possible explanation is that there are few or no persisters when these independent or mixed cultures are exposed to antibiotics and these phenotypically resistant bacteria are generated after the bacteria start replicating in the presence of these drugs. If this were the case and the persisters were able to replicate at a low rate or somehow be able covert non-persisters into persisters so as a population they have a net growth rate in the presence of the antibiotic, results similar to those observed can be generated.

To illustrate the plausibility of this hypothesis in a necessarily quantitative way, we use the semi-stochastic model presented in our opening “straw person” theoretical consideration, but assume the probability of producing persisters, *fe* and *fs* is low (10^−7^ per cell per hour or less) and that in presence of the drug, the antibiotic refractory persisters have a maximum rate of increase of 0.2 per cell hour. The results of these simulation experiments are presented in Table 2 for four groups of 10 independent runs of antibiotics with different pharmacodynamics (Hill function parameters) and/or initial concentrations. Two related results are particularly note-worthy. First, the ratio of the standard deviation to mean number of persisters is similar to that observed in our experiments. Second, as also observed in our experiments, a number of runs terminated without persisters being detected.

**Table 2:** Simulated persister generation experiments.

MIC	V_MIN_	K_a_	fe = fs	A	Mean	STDEV	Zeros
1	−3	1	5.00E-07	100	143.0	118.8	3
					185.4	179.1	2
					246.7	180.6	2
					144.5	92.3	2
1	−1	1	1.00E-07	10	130.9	125.7	2
					103.1	117.2	5
					151.1	150.7	2
					115.1	65.8	1
0.5	−0.5	0.5	5.00E-08	100	88.3	85.1	3
					78.6	69.5	2
					74.5	73.9	4
					68.5	80.5	4

Mean and standard deviation in the number of persisters for 10 runs and number of the 10 runs with no persisters recovered (Zeros). The initial number of cells is identical for all sets of simulation. Four groups of 10 independent runs with different antibiotic pharmacodynamics, persister generation rates and/or initial concentrations.

It should be emphasized that the preceding model is probably not the only mechanism that could account for the high variance between cultures in the number of persisters, the zero classes, and why mixtures of independent cultures generate similar results as independent cultures. As one reviewer of an earlier draft of this report suggested, it could well be that the different types of persisters are generated in these cultures and these vary in their ability to form colonies when exposed to antibiotics on the selecting agar. Either way, these results clearly suggest that the antibiotics play a role in determining how many persisters are produced.

#### Test of this hypothesis

**Figure 6:**
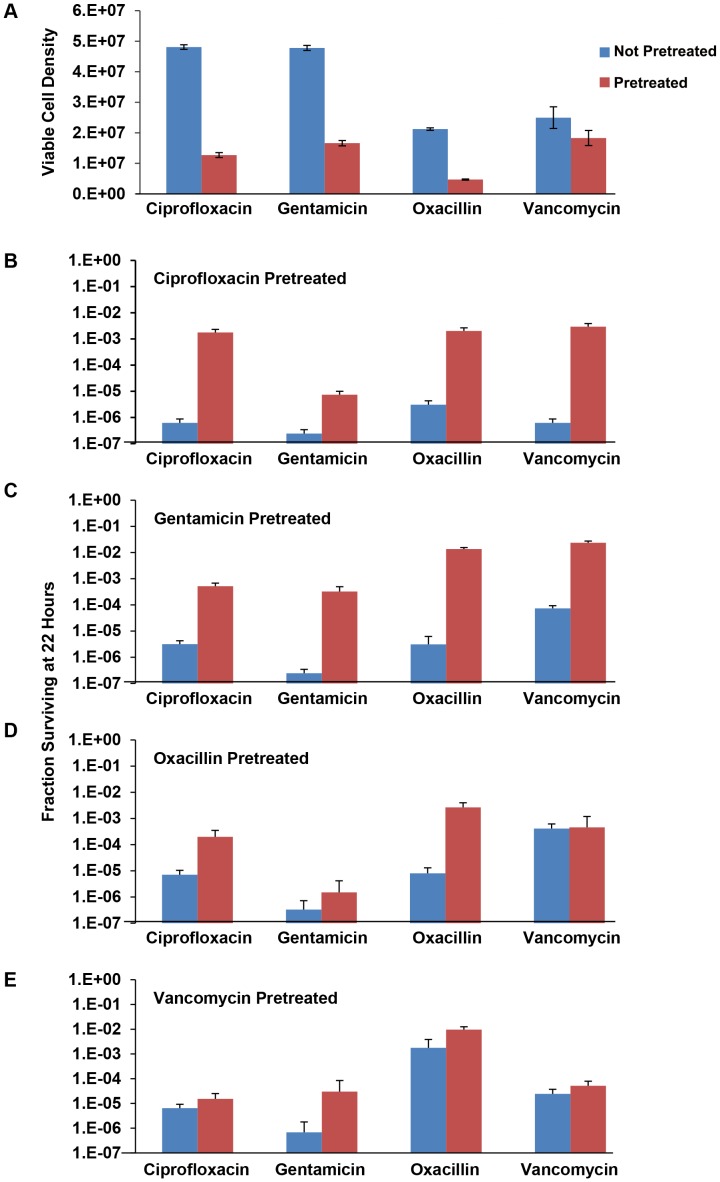
Effects of pre-exposure to sub-MIC drug concentrations on persistence levels. (A) Initial density of the 24 hour unexposed and antibiotic pre-treated bacteria. (B) Fraction of surviving cells (persisters) of *S. aureus* pre-treated with 0.2× MIC ciprofloxacin and unexposed controls after secondary treatment with 20× MIC of indicated antibiotic for 22 hours. (C) Fraction of surviving cells (persisters) of *S. aureus* pre-treated with 0.2× MIC gentamicin and unexposed controls after secondary treatment with 20× MIC of indicated antibiotic for 22 hours. Each data point represents the mean and standard error for 10 independent cultures. (D) Fraction of surviving cells (persisters) of *S. aureus* pre-treated with 0.5× MIC oxacillin and unexposed controls after secondary treatment with 20× MIC of indicated antibiotic for 22 hours. Each data point represents the mean and standard error for 10 independent cultures. (E) Fraction of surviving cells (persisters) of *S. aureus* pre-treated with 0.2× MIC vancomycin and unexposed controls after secondary treatment with 20× MIC of indicated antibiotic for 22 hours. Each data point represents the mean and standard error for 10 independent cultures.

If, as assumed above, in some way antibiotics promote the generation and/or ascent of persisters, then bacteria cultured in media with sub-MIC concentrations of antibiotics should have greater numbers of persisters than that found with unexposed bacteria. Most importantly, the number of persisters generated in these pre-treated cultures would be greater for all antibiotics, not only those to which the bacteria were pre-treated. Moreover, if this model is correct, there would be considerable between culture variations in the numbers of persisters. To test this hypothesis, we grew the bacteria with 0.2× MIC ciprofloxacin, gentamicin, vancomycin, or 0.5× MIC oxacillin and antibiotic free controls. Using the protocol employed in the preceding time-kill persister experiments, we then exposed these cultures to 20× MIC of each of the four antibiotics. The results of this experiment are presented in Figure 6.

Although there was no evidence for difference in the turbidity of the 24-hour cultures grown in 0.2× MIC ciprofloxacin, gentamicin, and vancomycin the CFU estimates of densities were significantly lower than the corresponding controls. The turbidity of the 24-hour 0.5× MIC pre-treated oxacillin cultures however, were lower than the control cultures as were the corresponding CFU estimates. Pre-treatment with sub-MIC concentrations of ciprofloxacin and gentamicin resulted in substantial increases in the fraction of surviving cells (level of persistence) when exposed to all four antibiotics. The effects of pre-exposure to oxacillin and vancomycin, however, depended on the antibiotic used for treatment. Pre-treatment with sub-MIC concentrations of oxacillin resulted in substantial increase in the level of persistence in cultures exposed to ciprofloxacin and oxacillin but not gentamicin or vancomycin. Pre-treatment with vancomycin only augmented the level of persistence in cultures treated with gentamicin. This experiment has been performed at least twice for all the pre-treatment and treatment drugs and the same results obtained.

It should be noted that in addition to these effects of pretreatment on the level of persistence the variance in the number of persisters among cultures for the pre-exposed populations appeared to be as great as that observed earlier. That is, the ratio of the standard deviation to the mean of surviving cells in the pre-treated cultures was similar to that of the controls that were treated but not pre-treated (data not shown).

## Discussion

In accord with the prevailing view, persisters are non- or slowly- growing cells generated at random from a dominant population of antibiotic susceptible bacteria. By killing growing cells, bactericidal antibiotics reveal the presence of these already existing genetically susceptible but phenotypically resistant subpopulations, but play no role in their generation and ascent. This perspective on the role of antibiotics in the generation and ascent of persisters was recently challenged by Dörr and colleague's demonstration that pre-exposure of *E. coli* to sub-MIC concentrations of the fluoroquinolone ciprofloxacin increases the frequency of persisters in cultures subsequently exposed to bactericidal concentrations of this drug [29]. The results presented in this jointly theoretical and experimental study with the Gram positive pathogen, *Staphylococcus aureus* add considerable generality to this observation. In their *E. coli* experiments, the only drug used for pretreatment was ciprofloxacin. Moreover the demonstration of an augmented level of persistence was also limited to a single drug, ciprofloxacin. In our experiments, pre-treatment to sub-MIC concentrations gentamicin as well as ciprofloxacin, oxacillin, and vancomycin increased the level of persistence to at least one of the four drugs used for treatment and not solely the drug used for pre-treatment. Indeed, pretreatment with ciprofloxacin and gentamicin substantially increased the level of persistence to all four drugs.

Further evidence in support of the hypothesis that antibiotics play a role in the production or ascent of persisters is the high variance in the fraction of persisters observed when independent cultures are exposed to antibiotics and the similarly high variances when mixtures of independent cultures are exposed to antibiotics. The logic behind this interpretation is the same as that which Luria and Delbruck employed [36] to distinguish between the random generation (pre-existence) of mutants and their production due to exposure to the agent selecting for their ascent. If the persisters were present before the cultures were exposed to antibiotics, contrary to what we observed there would be little variation in the number of persisters among the cultures derived from mixtures of the independent cultures. It should be noted, a high variance in the number of persisters in antibiotic treated cultures can be seen in, but was not remarked upon, earlier studies of persistence; for example, see the standard errors in Table 1 of the study by Wiuff and colleagues [20], and the error bars in Figure 1 of the investigation by Allison and collaborators [37].

In addition to providing generality and support for the proposition that antibiotics contribute to the generation and/or ascent of persisters, we also postulate and provide theoretical support for a mechanism by which antibiotics can play this role. The essence of this hypothesis is that there are few if any persisters present before the bacteria are exposed to these drugs. However, when exposed persisters are produced at a very low rate (on the order of 10^−8^ to 10^−7^) per cell per hour and, in the presence of antibiotics persisters either replicate at a low rate and/or in some way convert surviving non-persisters into persisters, see for example [38]. This mechanism however only accounts for the phenomenon of the antibiotic-associated generation of persistence at a population dynamic level. It is not founded on any specific physiological, molecular or genetic process for generating these phenotypically antibiotic resistant cells. Physiological and genetic mechanisms have, however, been postulated that could account for these results. For example, it is conceivable that these drugs induce stress responses that result in transient periods of arrested cell division [30], [39], or lead to unstable (and reversible) genetic changes, like amplification, which would then be subject to selection [40].

We do not consider the postulated population dynamic mechanism for antibiotics promoting the generation of persisters to be inconsistent with any of the hypothesis proposed for the physiological and molecular mechanisms responsible for the generation of these phenotypically- antibiotic refractory cells [24], [25], [41]–[49]. On the other hand, taken at large we believe our jointly theoretical and experimental results provide compelling support for the hypothesis that persistence can be attributed to many different mechanisms and that persisters are a diverse collection of different cell types [42].

That persistence is due to many different mechanisms [42], the majority of which are results of transient glitches and errors in the cell replication cycle or slow downs in cell metabolism, Persistence as Stuff Happens, PaSH, hypothesis can explain a variety of other observations. It provides at least two possible explanations for the different levels of persistence observed among the drugs used in the present experiments and other studies [37], [50]. First, as suggested earlier, a cell's entry into a transiently, replication- arrested, antibiotic-refractory state could be the product of a antibiotic induced stress responses [30]. The nature and extent of this stress response will almost certainly vary among drugs and their concentrations. Second, if, as suggested by [42], there are many different kinds of persister cells it is likely that the susceptibility of these bacteria to antibiotic-mediated killing would vary among drugs. An example of variation in the susceptibility of persisters to drugs has in fact been shown for some antimicrobial peptides [51].

Additional evidence in support of the PaSH hypothesis comes from essentially negative results. While there have been a number of attempts to identify the specific genes responsible for the persistence, it has not been possible to find mutants that fail to produce persisters in the surveys that have been conducted thus far [41], [52], [53], [54]. Also consistent with the PaSH hypothesis is the identification of large numbers of genes that increase or decrease frequency of persisters in different bacteria [54]. If persistence is due to different kinds of errors in cell division or reductions in rates of metabolism, it is not at all surprising that mutations in many different genes would alter the rates at which those errors occur.

### An evolutionary argument

Although not direct evidence in its support, the PaSH hypothesis provides a parsimonious explanation for the existence and ubiquity of persistence. In theory and experimentally, it has been shown that if bacterial populations are periodically challenged with agents that kill growing cells, “episodic selection” [55], a population that produces persisters can be favored in competition with an otherwise higher fitness population that does not produce them [25], [55]. However, the conditions under which episodic selection will favor populations that produce persisters are restrictive. Unless the persister producing population has an intrinsic advantage, when it is rare it will not be able to invade and become established in a population that does not produce persisters or produces them at a lower rate. The reason for this is that the phenotype favored by episodic selection, a low rate of the production of non- or slow growing cells, would not be manifest if the number of the persister-producing cells is low. For example, if there were 10^2^ persister-producing cells, *N*, in a population of 10^8^ non-producers, *M*, and persisters are produced at a rate of 10^−5^ per cell per hour, the probability that there will be a single persister cell is 10^−3^. Although random persisters may be generated, because of their low density, the *N* population is more likely to eliminated by a bactericidal antibiotic rather than generate persisters, which would allow for its survival.

For a more quantitative consideration of the conditions under which episodic selection will favor the evolution and maintenance of persistence, we use an extension of our opening model of the random production of persisters for bacterial populations maintained in continuous culture. We now consider two populations of bacteria, one that produces persisters, *P*, and one that does not respectively *N* and *M*. The *N*, *M* populations grow at a rate proportional to the concentration of a limiting resource, *R*, which is consumed at a rate proportional to the maximum growth rate of the bacteria and a conversion efficiency parameter, *e* µg. [58], [59]. The limiting resource from a reservoir where it is maintained at a concentration, *C* µg/ml flows into a habitat of unit volume (1 ml) at a constant rate, w per hour which is the same rate at which excess resource, antibiotics wastes and the bacterial population, *N*, *P* and *M* flow out. In this model we assume the persister, *P*, population does not replicate or take up the resource.

In this model, persisters are generated from *N* cells with a single probability *f* per cell per hour and revert back to the *N* state at a rate g per cell per hour. At each hour, there is a probability, *de*, that *A_MAX_* µg/ml of a bacteriocidal antibiotic will be added to the vessel. In addition to being washed out at a rate w per hour the effective concentration of the antibiotic can decay at a rate *da* per hour. As with the model described in the body of this report, we use a Hill function for the relationship between the concentration of the antibiotic and the rate of growth/death of the bacteria. We assume the same pharmacodynamics for the *N* and *M* populations and that the persister population is totally refractory to this drug.

In our simulation, the production of persisters and the return to the *N* state are stochastic processes as are incidence of episodes of antibiotic introduction is stochastic processes. For this use a Monte Carlo protocol. At each time interval, *Δt*, the probability that a persister cell will be generated is N*f*Δt, if a random number 0<*r*<1 is less than this product, a persister will be added to the *P* population and a single cell removed from the *N* population. The same protocol is used for conversion of *P* into *N*, but now the probability used for comparison is P*g*Δt. For these simulations we use values of *Δt* and other parameters such that at any time interval these products are less than 1. At each time interval a third random number is generated. If that number is less than de*Δt, A_MAX_ µg/ml of the antibiotic is introduced. The Berkeley Madonna program used for these is available from www.eclf.net/programs.

**Figure 7:**
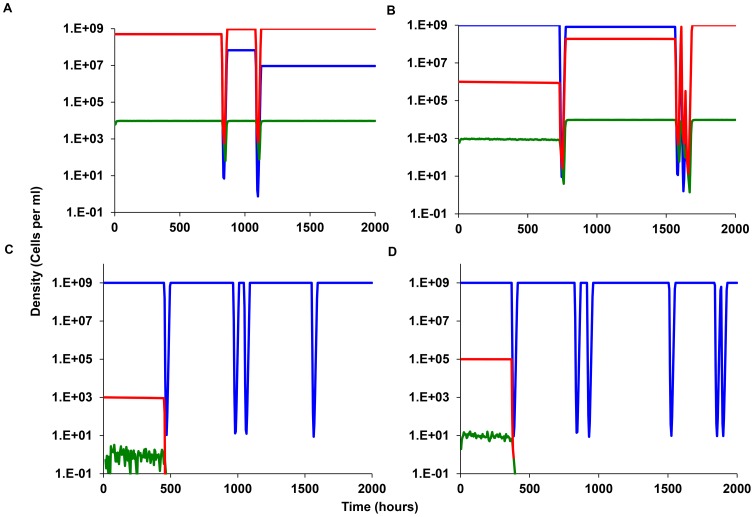
Simulation results, episodic selection for persistence in continuous culture. Bacteria that do and do not produce persisters, respectively *N* (red) and *M* (blue). The density of the persister population denoted by the green line. Common parameter values: *g* = 0.01, *V_MAX_ = 1.0, V_MIN_* = −2.0, *MIC* = 1, *κ* = 1, *epi* = 0.001, and *A_max_*_ = _50 µg/ml, *da* = 0.10 h_r-1_, *w* = 0.2 hr. for both *N* and *M*. Save for (D), f = 2×10^−4^ per cell per hour and in all simulations there are no persisters at time 0. (A) Changes in the density of *M (blue)* and *N* (Red) and the total persisters (Green) with initially equal densities (*N* = *M* = 5×10^8^). (B) Same as (A) but initially *N* = 10^6^ and *M* = 10^9^). (C) Same as (B) but with *N* = 10^3^ and *M* = 10^9^ initially. (D) Same as (C) but *f* = 2×10^−5^ per cell per hour.

For each of four sets of parameters, we made 20 independent simulations each for 2000 hours. In Figure 7A we present a single simulation where the *N* and *M* populations are initially present at the same density, *N = M* = 5×10^8^ (the chemostat equilibrium) and there are initially no persisters. At each episode of antibiotic treatment the viable cell density of the *N* and *M* populations decline as do that of the persisters. Although the latter are not killed by the antibiotics, their rate of production is reduced, due to the decline in *N* population and their reversion to the *N* state. They do, however, buffer the extent of decline of the *N* population and thereby provide the *N* cells an advantage, even though they grow at the same rate as the *M* population. This advantage of the *N* population also obtains when the initial frequency of *N* is somewhat lower than *M* (Figure 7B). However, when the persister producing *N* population is substantially rare than the *M* population (*N* = 10^3^ and *M* = 10^9^), the *N* population no longer has an advantage due production of antibiotic refractory persisters. Although persisters are generated by chance, their densities are too low to generate persisters and thereby prevent the elimination of the *N* population when confronted with the antibiotic. This threshold for density of *N* necessary for persistence to provide an advantage is even greater when the probability of producing persisters is lower. This can be seen in Figure 7D, where *f* = 10^−5^ rather than 10^−4^ per cell per hour. Under these conditions the persister-producing *N* population does not have an episodic selection advantage when its initial density is 10^5^ cells per hour. More details about the outcome of the 20 independent simulations with the four sets of parameters in Figure 7 are presented in Table 3.

**Table 3:** Outcomes of a series of 20 runs with the parameters in Figure 7.

	(A)	(B)	(C)	(D)
Number where M are lost* before 2000 hours	8	6	0	1
Mean time to loss of one population	849.4	1118.0	1118.0	579.6
Standard Deviation in the time to loss of one population.	457.1	360.1	475.7	480.9
Lowest time to loss of one population	60	675	40	15
Highest time of loss of one population	1505	1475	1900	1865
Number of populations where M>N at 2000 hours	0	0	20	15**

* By Loss we are considering situations where the density of one population becomes less than 0.5 cells per ml.

** In 5 simulated cultures neither *N* or *M* cells were present at 2000 hours.

In Figure 7A, 7B, and 7C, we follow the changes in density of the persister-producing *N* population and it's non- producing competitor *M*. In Figure 7A and 7B, if in addition to producing non-growing cells, the persister producing, *N* population has an intrinsic disadvantage in its maximum growth rate *V_MAXN_* = 0.995, *V_MAXM_* = 1.000. In the absence of episodic selection, the relative density of the *N* population declines (Figure 7A). When we allow for the random introduction of a decaying antibiotic, the production of non-growing persister cells can provides the otherwise less fit persister-producing *N* population with an advantage. In the simulation presented in Figure 7B the population that does not produce persisters, *M*, is lost by 3000 hours, whilst the persister-producing *N* population dominates. To get a better idea of the advantage to producing non-growing, antibiotic-refractory cells, we ran this simulation 50 times and calculated the *M/N* ratio at 1000 hours. In 39 out of the 50 runs, the ratio of *N/M* exceeded 1.0. If we assume a density of 0.1 cells per ml as the cut-off for the loss of a population, the average *N/M* ratio at 1000 hours for the 50 runs was 3.2×10^9^ with a standard deviation of 4.4×10^9^ cells per ml. The lowest *N/M* ratio noted for the 11 runs where the *M* dominated was 0.15.

A very different outcome is obtained when the persister-producing *N* population is initially rare and the production of persisters is a random process. Although this persister-producing population has an intrinsic growth rate advantage relative to the non-producing *M*, it is invariably lost when its initial frequency is low (Figure 7C). The rare N population is also most commonly lost when is initial numbers are somewhat higher, 10^5^ rather than 10^3^ (Figure 7D). The time before the loss of the rare persister-producing population (*N*<0.1 per ml) varies over a large range (Table 3). The reason for this is that at these low densities, antibiotic-refractory persister cells are rarely if ever produced. And, since this population with the capacity to produce persisters is rare, even though it has a higher intrinsic fitness, it is more likely to be eliminated by the antibiotic episodes than the more common non-persister producing *M*, population.

If the PaSH hypothesis is correct, and persisters are largely if not exclusively the product of errors in cell replication and cell metabolism, it is no more necessary to explain the evolutionary processes responsible for the existence of persisters than it is for mutation. Like mutation, persistence is an inevitable rather than an evolved character. The high frequency persister mutants, like *hipA* [56], [57], are analogous to the mutants with defective mismatch and other repair genes, “mutator” genes like *mutS*. As is the case for mutation, evolution by natural selection can favor mechanisms that alter the rate at which these errors occur but it cannot eliminate them any more than mutator genes eliminate mutation. Because persistence is an inevitable rather than an evolved character, we would expect it to be ubiquitous. Indeed, persistence has been shown to occur in single celled eukaryotes [17], [18] as well as prokaryotes [16] and the somatic cells responsible for neoplasms [19].

## Materials and Methods

### Bacterial strains, antibiotics, culture, and sampling media

The experiments herein utilize *Staphylococcus aureus* strain Newman which was originally isolated in 1952 from a patient suffering from tubercular osteomyelitis [32]. This strain was generously provided by Dr. William Shafer. Cultures were grown in 10 mL of Mueller-Hinton II (MHII) broth in 50 mL Pyrex flasks at 37°C shaking at 200 rpm. Viable cell density per ml was determined by dilution plating on Lysogeny Broth (LB) agar (also incorrectly, but commonly, known as Luria Bertani broth or Luria Broth) [33], [34]. Antibiotic stocks were prepared to a final concentration of 10 µg/µl for ciprofloxacin, gentamicin and oxacillin while vancomycin was prepared to a final stock concentration of 15 µg/µl. All antibiotics were procured from Mediatech, Inc. (Herndon, Va.) and Sigma-Aldrich (St. Louis, Mo.). Dilutions of requisite antibiotics were made fresh in MHII broth to the appropriate concentrations for each experiment.

### MICs and pharmacodynamic functions

The minimum inhibitory concentrations (MICs) of the antibiotics and bacteria used in this study were estimated with the factor of two serial dilution protocol using the method recommended by the Clinical and Laboratory Standards Institute (CLSI) [35]. In short, overnight cultures were grown in MHII, diluted to final concentrations of ∼10^5^ in fresh media and incubated 96 well plates with two-fold serial dilutions of each antibiotic. After incubation at 37°C for 18 hours, the OD_630 nm_ was obtained and the MICs estimated as the lowest concentration for which there was no growth. Using methods described in Regoes et al [31], we calculated the parameters of the pharmaocdymamic (Hill) functions for each combination of *S. aureus* and antibiotic.

### Time kill and persistence assay experiments

The bacteria were grown overnight in MHII broth with shaking (200 rpm) at 37°C in 50 mL Pyrex flasks. These overnight cultures were then diluted to a final concentration of ∼2×10^6^ or ∼2×10^7^ bacteria (depending on assay) in fresh MHII media and incubated for 1 hour at 37°C shaking at 200 rpm to ensure entry into the exponential growth phase. Cultures were then inoculated with different concentrations (multiples of the MICs) of ciprofloxacin, gentamicin, oxacillin, or vancomycin. Densities were estimated at different times by diluting and plating on LB agar followed by incubation at 37°C for 48 hours. To control for possible confounding by antibiotic carryover on the plates, experiments were performed with a washing protocol. The antibiotic-treated cultures were centrifuged at 5,000 rpm for 6 minutes. The pellets obtained were re-suspended in fresh MHII media and pelleted again at 5,000 rpm for 6 minutes. Following the second re-suspension in fresh MHII these twice-washed cells were either plated directly, or plated following dilution in saline. While the variance in numbers of colonies observed with the washed cells was similar to those plated without washing, the estimated densities were lower, presumably because of the loss of cells or their viability during pelleting. Consequently, we elected to plate the cultures without washing.

### Antimicrobial susceptibility testing

Disk diffusion (Becton, Dickinson, and Company) assays were used to test for changes in susceptibility following exposure to the antibiotics. For this, we grew individual colonies in MHII overnight and added 50 µl to 1.5 mL of LB Soft (top) agar which was then poured onto LB agar. Disks containing 5 µg ciprofloxacin, 10 µg gentamicin, 1 µg oxacillin, or 30 µg vancomycin were placed onto the plates and the plates were incubated overnight at 37°C. The following day, zones of inhibition were determined.

## Supporting Information

Figure S1 Two round short- term time kill assays. Changes in viable cell density for *S. aureus* Newman cultures treated with varying concentrations (0.5× MIC, MIC, 2.5× MIC, 5× MIC, and 10× MIC) of ciprofloxacin, gentamicin, oxacillin, and vancomycin are plotted. Following an initial round of time kill experiments, three individual surviving colonies were cultured overnight and each time kill assay pairing was repeated (ie surviving cells from ciprofloxacin 2.5× MIC treatment in the prior time kill assay were once more treated with 2.5× MIC ciprofloxacin) to evaluate killing dynamics of once treated cells. For the second round of time kill assays the average and standard deviations are plotted.
(PPT)

Text S1 Persister resistance is phenotypic not inherited. Central to the idea of persistence is that it is a transient form of phenotypic resistance. In this interpretation, when the surviving persister cells are re-cultured in absence of antibiotics and then exposed to drugs, they would behave as their naïve ancestors. To test this hypothesis we picked three colonies from *S. aureus* that had been exposed to supra- and sub-MIC concentrations of each of the four antibiotics studied. The re-cultured bacteria exposed to supra-MIC concentrations of all drugs had similar time kill dynamics as their naïve ancestors (Figure S1). Interestingly, the bacteria recovered from cultures exposed to sub-MIC concentrations of ciprofloxacin and gentamicin produced higher viable cell densities when exposed to sub-MIC concentrations of that drug. Since there was no evidence for increases in MIC or other indications of inherited, resistance, we assume that this too is a phenotypic effect of pre-exposure to antibiotics.
(DOCX)
