# Supplementary material for: Pharmacodynamics, Population Dynamics, and the Evolution of Persistence in Staphylococcus aureus
Source: PLoS Genet. 2013 Jan 3;9(1):e1003123. doi: 10.1371/journal.pgen.1003123 (PMC3536638; doi:10.1371/journal.pgen.1003123)
Supplement: Figure S1 — Two round short- term time kill assays. Changes in viable cell density for S. aureus Newman cultures treated with varying concentrations (0.5× MIC, MIC, 2.5× MIC, 5× MIC, and 10× MIC) of ciprofloxacin, gentamicin, oxacillin, and vancomycin are plotted. Following an initial round of time kill experiments, three individual surviving colonies were cultured overnight and each time kill assay pairing was repeated (ie surviving cells from ciprofloxacin 2.5× MIC treatment in the prior time kill assay were once more treated with 2.5× MIC ciprofloxacin) to evaluate killing dynamics of once treated cells. For the second round of time kill assays the average and standard deviations are plotted. (PPT) [file pgen.1003123.s001.ppt]

## Slide 1
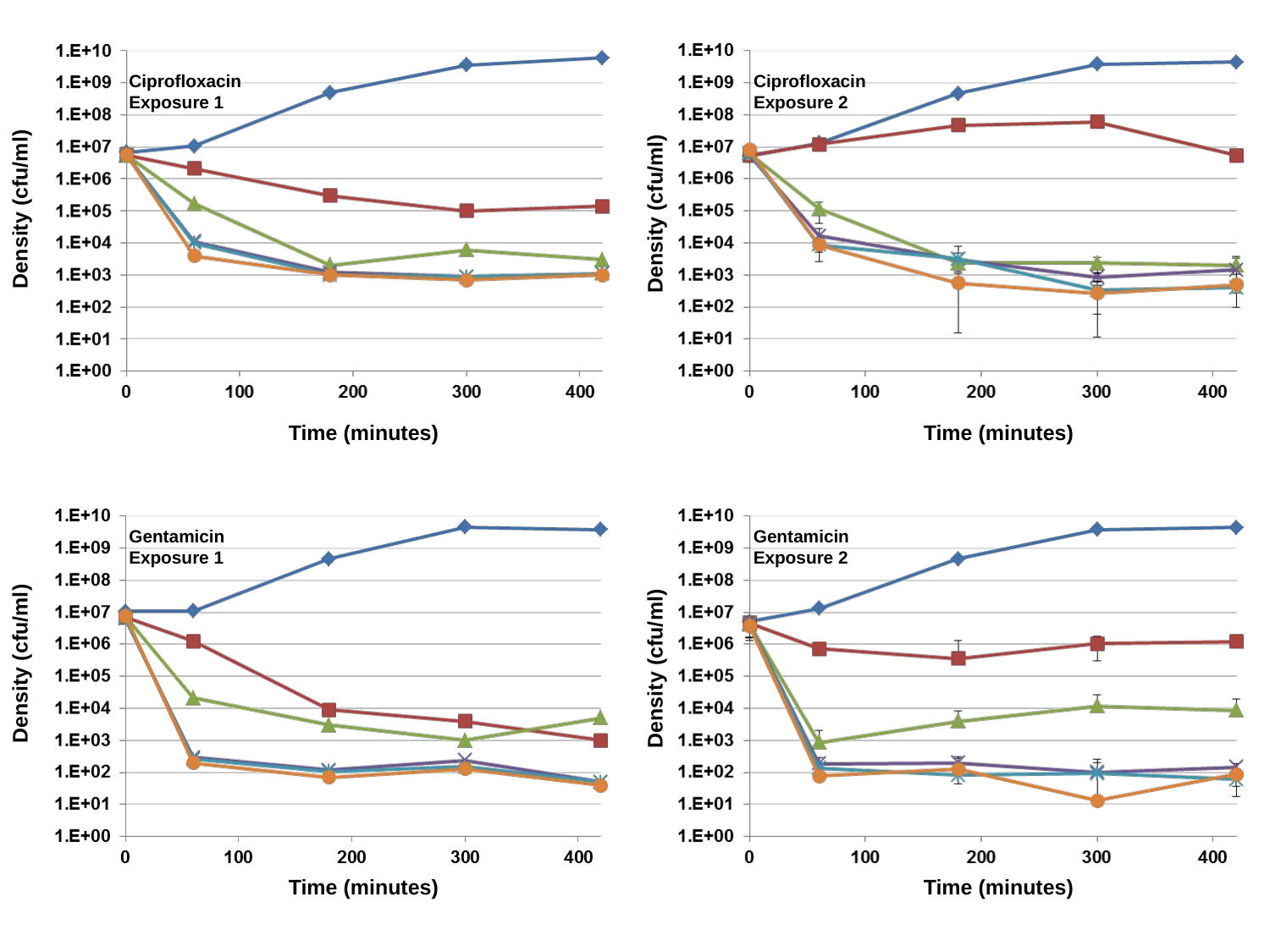

Ciprofloxacin
Exposure 1
Ciprofloxacin
Exposure 2
Density (cfu/ml)
Density (cfu/ml)
Time (minutes)
Time (minutes)
Gentamicin
Exposure 1
Gentamicin
Exposure 2
Density (cfu/ml)
Density (cfu/ml)
Time (minutes)
Time (minutes)

## Slide 2
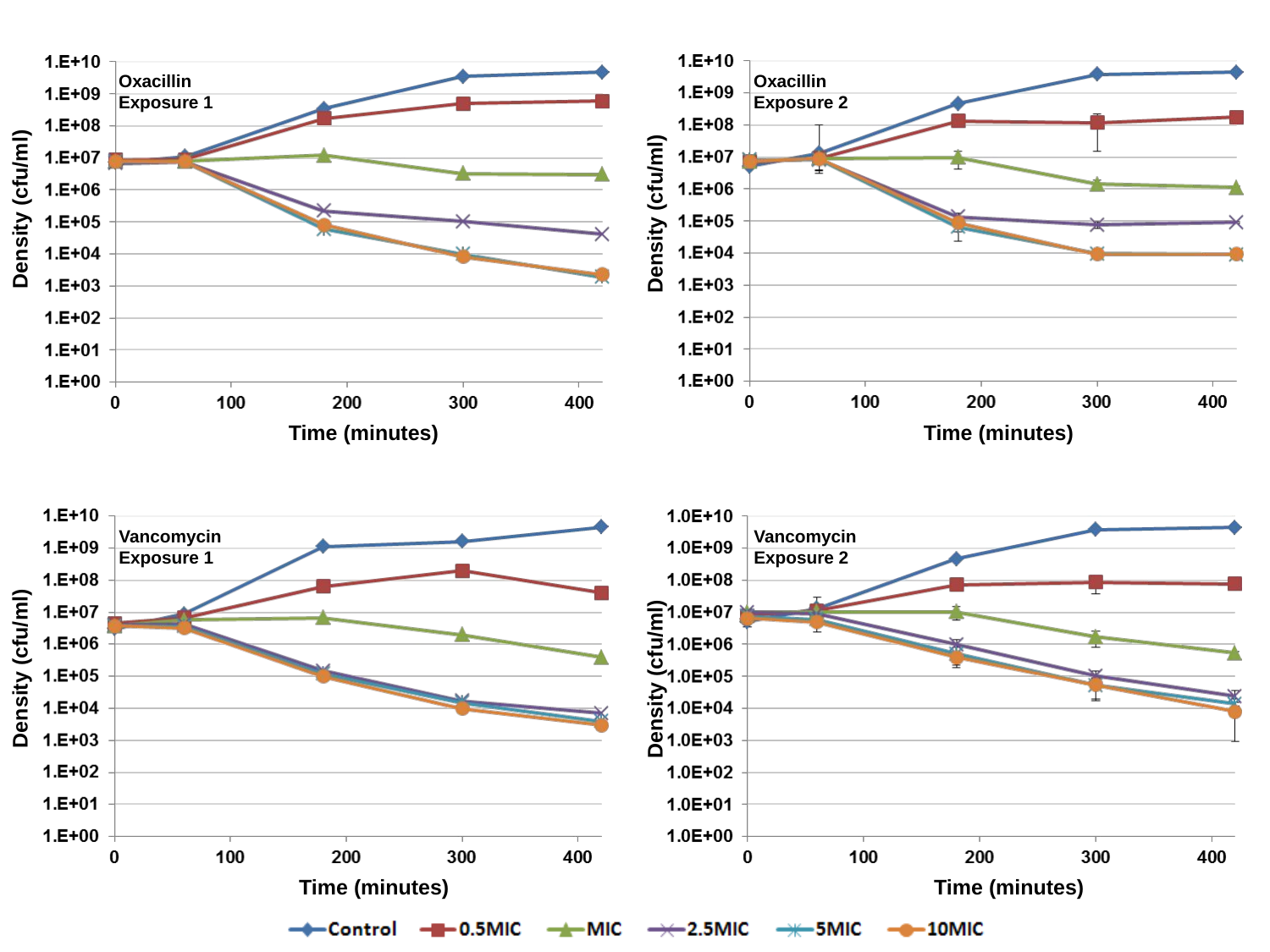

Oxacillin
Exposure 1
Oxacillin
Exposure 2
Density (cfu/ml)
Density (cfu/ml)
Time (minutes)
Time (minutes)
Vancomycin
Exposure 1
Vancomycin
Exposure 2
Density (cfu/ml)
Density (cfu/ml)
Time (minutes)
Time (minutes)
