# Supplementary material for: Pharmacodynamics, Population Dynamics, and the Evolution of Persistence in Staphylococcus aureus
Source: PLoS Genet. 2013 Jan 3;9(1):e1003123. doi: 10.1371/journal.pgen.1003123 (PMC3536638; doi:10.1371/journal.pgen.1003123)
Supplement: Text S1 — Persister resistance is phenotypic not inherited. Central to the idea of persistence is that it is a transient form of phenotypic resistance. In this interpretation, when the surviving persister cells are re-cultured in absence of antibiotics and then exposed to drugs, they would behave as their naïve ancestors. To test this hypothesis we picked three colonies from S. aureus that had been exposed to supra- and sub-MIC concentrations of each of the four antibiotics studied. The re-cultured bacteria exposed to supra-MIC concentrations of all drugs had similar time kill dynamics as their naïve ancestors (Figure S1). Interestingly, the bacteria recovered from cultures exposed to sub-MIC concentrations of ciprofloxacin and gentamicin produced higher viable cell densities when exposed to sub-MIC concentrations of that drug. Since there was no evidence for increases in MIC or other indications of inherited, resistance, we assume that this too is a phenotypic effect of pre-exposure to antibiotics. (DOCX) [file pgen.1003123.s002.docx]

**Text S1.**

Central to the idea of persistence is that it is a transient form of phenotypic resistance. In this interpretation, when the surviving persister cells are re-cultured in absence of antibiotics and then exposed to drugs, they would behave as their naïve ancestors. To test this hypothesis we picked three colonies from *S. aureus* that had been exposed to supra- and sub-MIC concentrations of each of the four antibiotics studied. The re-cultured bacteria exposed to supra-MIC concentrations of all drugs had similar time kill dynamics as their naïve ancestors (Figure S1). Interestingly, the bacteria recovered from cultures exposed to sub-MIC concentrations of ciprofloxacin and gentamicin produced higher viable cell densities when exposed to sub-MIC concentrations of that drug. Since there was no evidence for increases in MIC or other indications of inherited, resistance, we assume that this too is a phenotypic effect of pre-exposure to antibiotics.
